# Comparing reappraisal and acceptance strategies to understand the neural architecture of emotion regulation: a meta-analytic approach

**DOI:** 10.3389/fpsyg.2023.1187092

**Published:** 2023-07-21

**Authors:** Bianca Monachesi, Alessandro Grecucci, Parisa Ahmadi Ghomroudi, Irene Messina

**Affiliations:** ^1^Clinical and Affective Neuroscience Lab, Department of Psychology and Cognitive Sciences—DiPSCo, University of Trento, Rovereto, Italy; ^2^Center for Medical Sciences—CISMed, University of Trento, Trento, Italy; ^3^Universitas Mercatorum, Rome, Italy

**Keywords:** reappraisal, acceptance, emotion regulation processes, meta-analysis, activation likelihood estimation method

## Abstract

**Introduction:**

In the emotion regulation literature, the amount of neuroimaging studies on cognitive reappraisal led the impression that the same top-down, control-related neural mechanisms characterize all emotion regulation strategies. However, top-down processes may coexist with more bottom-up and emotion-focused processes that partially bypass the recruitment of executive functions. A case in point is acceptance-based strategies.

**Method:**

To better understand neural commonalities and differences behind different emotion regulation processes, in the present study, we applied the Activation Likelihood Estimation (ALE) method to perform a meta-analysis on fMRI studies investigating task-related activity of reappraisal and acceptance. Both increased and decreased brain activity was taken into account in the contrast and conjunction analysis between the two strategies.

**Results:**

Results showed increased activity in left-inferior frontal gyrus and insula for both strategies, and decreased activity in the basal ganglia for reappraisal, and decreased activity in limbic regions for acceptance.

**Discussion:**

These findings are discussed in the context of a model of common and specific neural mechanisms of emotion regulation that support and expand the previous dual-routes models. We suggest that emotion regulation may rely on a core inhibitory circuit, and on strategy-specific top-down and bottom-up processes distinct for different strategies.

## Introduction

In affective neuroscience and clinical psychology, emotion regulation (Gross, 1998) has emerged as a core construct widely applied to the conceptualization of neurobiological models of affective disorders (Taylor and Liberzon, 2007; Kring and Sloan, 2009; Grecucci et al., 2020; Messina et al., 2021) and their treatment (Beauregard, 2007; Messina et al., 2013; Grecucci et al., 2015, 2017; Frederickson et al., 2018). Alongside this growing scientific interest in emotion regulation, there has been a rising debate regarding the usefulness of different emotion regulation strategies and their implications for therapeutic techniques (Leahy et al., 2011; Wolgast et al., 2011; Dadomo et al., 2016, 2018; Grecucci et al., 2017). In this debate, reappraisal and acceptance are often mentioned as effective strategies for regulating emotions and mechanisms of psychotherapy action (Wolgast et al., 2011; Grecucci et al., 2020; Spencer et al., 2020).

Reappraisal is defined as “*construing a potentially emotion-eliciting situation in non-emotional terms*” (Gross, 2002, p. 281). It has been traditionally deemed adaptive, since associated with reduced neuropsychological response to emotional events (e.g., Kanske et al., 2012; Webb et al., 2012), and with general well-being and mental health (Aldao et al., 2010). Reappraisal strategy allows individuals to change the appraisals that contribute to negative emotions (Gross, 1998), by highly engaging cognitive resources as reflected in the involvement of a complex pattern of prefrontal cortical regions (Ochsner and Gross, 2005). Reappraisal is clearly related to traditional cognitive behavioral therapy (CBT), which uses cognitive restructuring to alleviate psychological suffering by changing how the patients interpret and think about their everyday experiences (Beck et al., 1979). We acknowledge that different types of reappraisal strategy exist (i.e., reinterpretation and distancing), and that previous studies have highlighted that they rely on partial distinct mechanisms and cortical brain areas (Messina et al., 2015; Powers and LaBar, 2019). In this study, we will focus only on the reinterpretation strategy, referred to as reappraisal hereafter, and intended as the reappraised situation or the cause of the stimulus, without any change in the perspective taken.

On the other hand, acceptance can be described as a mental stance toward ongoing mental and sensory experiences, characterized by openness, curiosity, and non-evaluative attitude (Grecucci et al., 2015; Goldin et al., 2019). It involves the recruitment of very few cognitive resources and relies on prefrontal cortical areas (Messina et al., 2021). Acceptance is the core of the so-called third-wave behavioral therapies (Hayes, 2004; Kahl et al., 2011). In this context, it has been described as “*the active and ware embrace of private experiences without unnecessary attempts to change their frequency or form*” (Hayes et al., 2012, p.982) and it is taught as the counter of experiential avoidance. Implicitly, psychodynamic and humanistic approaches also work on experiential avoidance/acceptance, encouraging the experience of emotions and the associated physical impulse in the body rather than down-regulating them through cognitive or attentional mechanisms (Frederickson et al., 2018; Messina et al., 2020, 2021).

In terms of psychophysiological effects, both reappraisal and acceptance are widely considered adaptive strategies (Aldao et al., 2010). Previous studies that have experimentally compared these strategies have reported their effectiveness in reducing experimentally inducted negative emotions and physiological activation, although slight differences have emerged. When comparing their efficacy in reducing short-term negative emotions, reappraisal was generally found to be slightly superior to acceptance in most cases (Hofmann et al., 2009; Szasz et al., 2011; Smoski et al., 2015; Troy et al., 2018; Goldin et al., 2019), although other studies found no significant differences (Wolgast et al., 2011; Asnaani et al., 2013). Regarding physiological reactivity, Hofmann et al. (2009) reported similar effectiveness of both strategies in decreasing heart rate, compared to suppression. Goldin et al. (2019) found no difference in respiration rate and skin conductance, but higher heart rate in reappraisal compared to acceptance. Wolgast et al. (2011) found that reappraisal was slightly more effective than acceptance at reducing skin conductance, whereas Troy et al. (2018) reported the opposite result. Finally, only one study (Troy et al., 2018) examined the perceived cognitive costs of using these two strategies, reporting that acceptance was perceived as less difficult to employ than reappraisal.

Although these results suggest that both reappraisal and acceptance can be considered useful strategies, the underlining neurobiological mechanisms are still poorly understood. Investigating the common and different brain regions associated with reappraisal and acceptance may not only clarify their specific nature but also unveil the control-related brain areas underlying top-down vs. bottom-up (emotion focused) strategies, thereby contributing to a deeper understanding of the mechanisms of emotion regulation. Traditional models of emotion regulation are largely based on, and overlap with, the neural structures involved in reappraisal (Ochsner and Gross, 2005), despite the growing body of evidence on more emotion-focused regulation modalities (Messina et al., 2021). A recent study (Messina et al., 2021) has pointed out that its neural correlates of acceptance may differ from those of reappraisal, with a less clear relevance of prefrontal control brain areas and possibly involving more bottom-up mechanisms. Unfortunately, this study did not report a direct comparison between acceptance and reappraisal, leaving the possible differences between the two strategies speculative.

To accommodate this emerging literature, some authors have proposed a dual-route model for emotion regulation, suggesting the possibility of different top-down cognitive control mechanisms and bottom-up emotion focused mechanisms (e.g., Grecucci et al., 2020). However, dual-route models may be simplistic, and an intriguing hypothesis is that there might also be a common mechanism underlying different strategies (Morawetz et al., 2017). To date, only four task-based fMRI experiments have directly compared reappraisal and acceptance (Opialla et al., 2015; Smoski et al., 2015; Goldin et al., 2019; Dixon et al., 2020). In most of these studies, greater brain responses in prefrontal brain regions implicated in cognitive control, such as the dorso-lateral prefrontal cortex (DLPFC) and dorso-medial prefrontal cortex (DMPFC) have been observed in reappraisal compared to acceptance (Smoski et al., 2015; Goldin et al., 2019; Dixon et al., 2020). Some studies have also associated acceptance with reduced activity in parts of the default mode network (DMN) (Opialla et al., 2015; Dixon et al., 2020). The DMN is a set of areas that are anti-correlated with executive processes and associated with mind-wandering (Christoff et al., 2009). Since mind-wandering has been considered as the opposite of mindfulness (Mrazek et al., 2012), these effects on DMN have been interpreted as interruptions of ruminative, self-reflective processes related to emotions, which are independent from executive processes (Ellard et al., 2017; Messina et al., 2021). Additionally, Dörfel et al. (2014) reported greater activation in regions linked to somatic and emotion awareness (left insular cortex and right prefrontal gyrus) in acceptance compared to reappraisal. In other words, these studies suggest that reappraisal and acceptance may rely on different neural substrates: reappraisal involves a regulatory mechanism based on cognitive control and supported by prefrontal executive regions, while acceptance operates without the involvement of executive areas and is based on the reduction of brain activity in subcortical areas and the DMN. However, a few experiments have reported increased prefrontal activations for acceptance (Lebois et al., 2015; Goldin et al., 2019). Therefore, it possible that a common core mechanism exists independently of the specific strategy used.

To provide evidence on this issue, the present meta-analytic study aimed to compared fMRI studies of reappraisal and acceptance in order to shed light on the possible common and distinct neural mechanisms underlying them. By doing so, these results may also offer insight into the potential mechanisms involved in these two types of strategies. Reappraisal-based strategies have traditionally been regarded as relying on control-related or “top-down” regulation mechanisms, while acceptance-based strategies have been conceptualized as relying on emotion focused or “bottom-up” regulation mechanisms (Grecucci et al., 2020; Messina et al., 2021). Demonstrating that these two strategies rely on different neural mechanisms may suggest that they rely on different psychological mechanisms too.

In the present study, our objective is to explore this hypothesis using a coordinate-based Activation Likelihood Estimation (ALE) method (Laird et al., 2005). This method allows to quantitatively compare two sets of functional Magnetic Resonance Imaging (fMRI) studies that have examined whole-brain activity during reappraisal and/or acceptance conditions relative to a baseline control condition where no regulation was performed. We employed a conjunction analysis to identify potential core common regulation mechanisms involved in both strategies. Additionally, a contrast analysis was conducted to identify significant clusters of brain activity that are specific of each of the two strategies. In both the conjunction and contrast analysis, we examined regions showing increased and decreased activity.

Previous meta-analytic studies on emotion regulation strategies have been conducted, especially on reappraisal (Buhle et al., 2014; Frank et al., 2014; Messina et al., 2015; Morawetz et al., 2017). They consistently found increased activity in prefrontal areas typically related to top-down control, such as DLPFC and DMPFC. Interestingly, previous effort to meta-analytically contrast reappraisal with other emotion regulation strategies highlighted some common regions, among which insula and VLPFC (Messina et al., 2021: Morawetz et al., 2017). However, it is still difficult to establish a clear understanding of the different activations for top-down and bottom-up emotion regulation strategies. Previous studies mainly focused on the contrast between reappraisal and other top-down strategies (e.g., distraction, Buhle et al., 2014), or combined reappraisal with very different strategies in their sample (e.g., mindfulness and suppression, Morawetz et al., 2017). Therefore, we believe our approach has the potential to unravel how bottom-up emotion regulation strategies may represent a different class from top-down strategies, despite both being adaptive and successful ER processes.

On the basis of previous meta-analyses, we hypothesize that the ventro-lateral prefrontal cortex (VLPFC) and insula may be confirmed as good candidates for a core common mechanism due to their strategic position in inhibiting emotion related areas and their implication in language functions (semantic and phonological ones), particularly the left hemisphere. Additionally, we believe that reappraisal-based strategies may engage the large dorso-lateral portions of the prefrontal cortex (Buhle et al., 2014), while acceptance-based strategies may involve subcortical limbic structures (Messina et al., 2021).

## Methods

### Study selection

**Figure 1:**
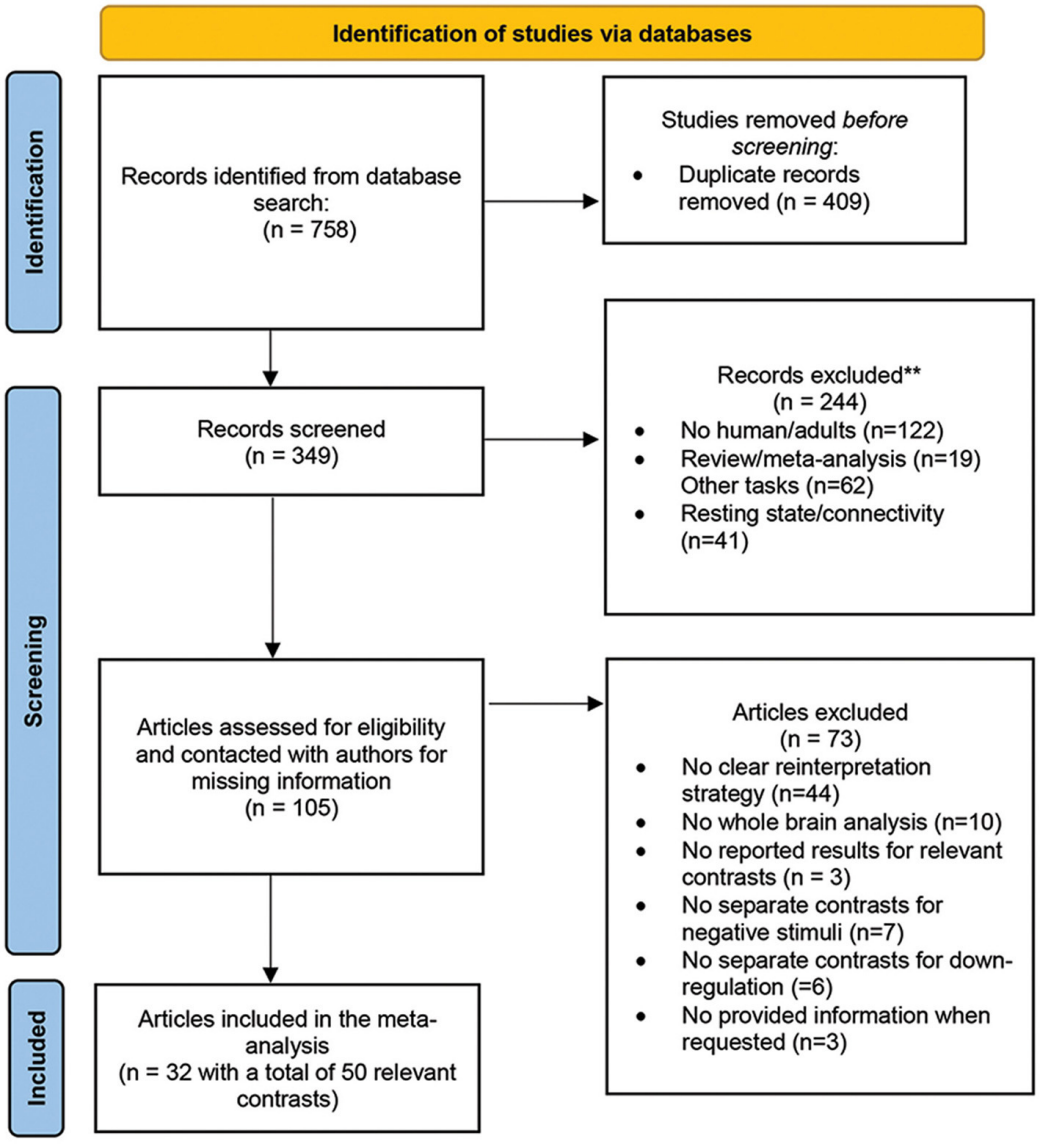
Flowchart of the literature search and study selection process, based on PRISMA template (Page et al., 2021).

The authors conducted a systematic online search on PubMed (https://www.ncbi.nlm.nih.gov/pubmed) and Google scholar (https://scholar.google.com) up until August 2022 to select the studies. The search used the keywords such as “emotion regulation,” “emotion regulation strategies” AND “reappraisal,” “acceptance” and/or “mindfulness” AND “fMRI” or “neuroimaging.” The references of retrieved studies as well as relevant previous reviews, systematic reviews and meta-analyses, were also hand-searched for additional supplementation. The entire screening process followed the PRISMA guidelines (Page et al., 2021) and the PRISMA 2020 checklist (see Supplementary Table 1 and Figure 1 for the PRISMA flowchart). No previous registration or protocol was prepared.

In the initial selection process, we included studies that employed the typical emotion regulation task, where a condition of emotion regulation was compared to a control condition of no-regulation during the presentation of emotional stimuli. The inclusion criteria for this selection were as follows:

- studies that reported specific contrasts of emotion regulation (acceptance/reappraisal) > no-regulation and/or the no-regulation > emotion regulation (acceptance/reappraisal);
- studies that performed univariate whole-brain analysis (studies or analysis using ROI approach were excluded to avoid inflated results, Müller et al., 2018);
- studies that reported Montreal Neurological Institute (MNI) and Talairach coordinates were reported;
- studies that included only on adult participants aged between 18 and 55 years, who were drug-free and had no neurological diseases.

The authors applied exclusion criteria to the retrieved studies, which were as follows: (i) studies with unclear or not specific reinterpretation strategy, such as reappraise the situation or the cause of the stimulus, without any change in perspective taking (e.g., distancing strategy); (ii) studies that did not provide a separate contrast for negative stimuli; (iii) studies that did not provide a separate contrast for down-regulation; (iv) studies that did not report significant foci (see Müller et al., 2018 for the sensitivity of coordinate-based algorithm to non-significant results), and (v) studies that did not provide information when requested. For an overview of the specific instructions used in the acceptance studies (see Messina et al., 2021). The final dataset included 32 studies that investigated acceptance and/or reappraisal. Studies with more than one relevant contrast, or separate analysis between conditions or participants groups were considered as independent samples, resulting in a total of 50 records included in the meta-analysis (see Table 1).

**Table 1:** Studies included in the meta-analysis.

**Studies**		**Contrast**	***N***	**Age M(SD)**	**N foci**
1	Lutz et al. (2014)	Acc vs. no-regulation	24	29.98 (7.96)	3
2	Smoski et al. (2015)	Acc vs. no-regulation	19 (12 F)	27.9 (6.3)	8
3	Smoski et al. (2015)	Acc vs. no-regulation	19 (12 F)	27.9 (6.3)	5
4	Murakami et al. (2015)	Acc vs. no-regulation	21 (11 F)	25.1 (5.5)	22
5	Lebois et al. (2015)	Acc vs. no-regulation	30 (15 F)	18–23	8
6	Ellard et al. (2017)	Acc vs. no-regulation	21 F	29.48 (8.44)	2
7	Goldin et al. (2019)	Acc vs. no-regulation	35 (57% F)	32.2 (8.9)	11
8	Dixon et al. (2020)^*^	Acc vs. no-regulation	113 (61 F)	32.9 (7.92)	2
9	Kross et al. (2009)	No-regulation vs. acc	24 (15 F)	20.83 (3.27)	64
10	Lutz et al. (2014)	No-regulation vs. acc	24	29.98 (7.96)	2
11	Lebois et al. (2015)	No-regulation vs. acc	30 (15 F)	18–23	1
12	Ellard et al. (2017)	No-regulation vs. acc	21 F	29.48 (8.44)	10
13	Kober et al. (2019)	No-regulation vs. acc	17 (5 F)	31.75 (5.18)	3
14	Kober et al. (2019)	No-regulation vs. acc	17 (5 F)	31.75 (5.18)	9
15	Goldin et al. (2019)	No-regulation vs. acc	35 (57% F)	32.2 (8.9)	4
16	Dixon et al. (2020)^*^	No-regulation vs. acc	113 (61 F)	32.9 (7.92)	9
17	Dixon et al. (2020)^*^	No-regulation vs. acc	35 (22 F)	32.1 (8.70)	6
18	Westbrook et al. (2013)	No-regulation vs. acc	48 (31% F)	45 (11.35)	1
19	Che et al. (2015)	Reap vs. no-regulation	29 (15 F)	22.62 (1.59)	8
20	Dixon et al. (2020)^*^	Reap vs. no-regulation	35 (22 F)	32.1 (8.70)	19
21	Dörfel et al. (2014)	Reap vs. no-regulation	19 F	18−39	17
22	Fitzgerald et al. (2020)	Reap vs. no-regulation	49 (67% F)	25.24 (7.98)	13
23	Gianaros et al. (2014)	Reap vs. no-regulation	157 (88 F)	30–54	21
24	Goldin et al. (2008)	Reap vs. no-regulation	17 F	22.7 (3.5)	18
25	Goldin et al. (2019)	Reap vs. no-regulation	35 (57% F)	32.2 (8.9)	13
26	Golkar et al. (2012)	Reap vs. no-regulation	58 (32 F)	24.02 (2.26)	11
27	Harenski and Hamann (2006)	Reap vs. no-regulation	10 F	18–29	7
28	Herwig et al. (2007)	Reap vs. no-regulation	18	23–36	2
29	Macdonald et al. (2020)	Reap vs. no-regulation	19	27	8
30	Morawetz et al. (2016a)	Reap vs. no-regulation	59 (20 F)	32.47 (11.25)	2
31	New et al. (2009)	Reap vs. no-regulation	14 F	31.7 (10.3)	14
32	Ochsner et al. (2002)	Reap vs. no-regulation	15 F	21.9	12
33	Qu and Telzer (2017)	Reap vs. no-regulation	29 (14 F)	19.2	11
34	Silvers et al. (2015)	Reap vs. no-regulation	30 (13 F)	21.97	48
35	Simsek et al. (2017)	Reap vs. no-regulation	15 F	22.53 (1.80)	8
36	van der Velde et al. (2015)	Reap vs. no-regulation	51 (47 F)	37.1 (10.3)	21
37	Vanderhasselt et al. (2013)	Reap vs. no-regulation	42 F	21.26 (2.29)	7
38	Wager et al. (2008)	Reap vs. no-regulation	30 (18 F)	22.3	8
39	Wu et al. (2019)	Reap vs. no-regulation	15	21-−27	10
40	Yoshimura et al. (2014)	Reap vs. no-regulation	15 (9 F)	23.3 (2.2)	7
41	Ziv et al. (2013)	Reap vs. no-regulation	27 (12 F)	31.1 (7.6)	11
42	Ziv et al. (2013)	Reap vs. no-regulation	27 (12 F)	31.1 (7.6)	1
43	Gianaros et al. (2014)	No-regulation vs. reap	157 (88 F)	30–54	17
44	Goldin et al. (2019)	No-regulation vs. reap	35 (57% F)	32.2 (8.9)	1
45	Herwig et al. (2007)	No-regulation vs. reap	18	23–36	3
46	Koenigsberg et al. (2010)	No-regulation vs. reap	16 (9 F)	31.8 (7.7)	5
47	Kross et al. (2009)	No-regulation vs. reap	24 (15 F)	20.83 (3.27)	5
48	Macdonald et al. (2020)	No-regulation vs. reap	19	27	5
49	Qu and Telzer (2017)	No-regulation vs. reap	29 (14 F)	19.2	2
50	Yoshimura et al. (2014)	No-regulation vs. reap	15 (9 F)	23.3 (2.2)	3

F, female; acc, acceptance strategy; reap, reappraisal strategy; M, Mean; SD, Standard Deviation. Multiple contrasts in each article are reported separately. (^*^) means data provided by authors on request.

### ALE analyses procedure

The Activation Likelihood Estimation (ALE) method (Eickhoff et al., 2009) is based on an algorithm that is able to overcome spatial uncertainty associated with neuroimaging studies. This method treats each focus coordinates as the center of a Gaussian spatial probability distribution. The resulting ALE maps consist in the spatial convergence of activation probabilities across foci from different experiments. To distinguish true convergence from random clustering, a permutation procedure is applied (Eickhoff et al., 2009). The GingerALE v3.02 software (http://brainmap.org/) was used for all analyses in this study.

Before performing the conjunction and contrast analyses, all foci were converted in MNI coordinates using icbm2tal transform (Lancaster et al., 2007). Separate ALE analyses were then performed on the following subsets: (a) reappraisal vs. no-regulation, to obtain the ALE map of increased brain activity in reappraisal; (b) no-regulation vs. reappraisal, to obtain the ALE map of decreased brain activity in reappraisal; (c) acceptance vs. no-regulation, to obtain the ALE map of increased brain activity in acceptance; (d) no-regulation vs. acceptance, to obtain the ALE map of decreased brain activity in acceptance. For each separate analysis, statistical significance was assessed and corrected for multiple comparisons using a cluster-level family-wise error method (FEW, Eickhoff et al., 2016; Müller et al., 2018), with a threshold of *p* < 0.05. Additionally, an uncorrected cluster-forming threshold of *p* = 0.01, and 1000 permutations were used.

After obtaining the four ALE images, a contrast analysis and a conjunction analysis were computed between the acceptance/reappraisal and no-regulation subsets (map of increased activity), and between the no-regulation and acceptance/reappraisal subsets (map of decreased activity). The contrast analysis involved subtracting one ALE image from the other, resulting in two ALE contrast images. The conjunction analysis aimed to identify the overlap or similarity between the datasets by using the voxel-wise minimum value of the ALE images. To account for study size variations, a study size correction method (Eickhoff et al., 2012) was applied using GingerALE. The foci datasets were randomly divided into two groups of the same size as the original datasets, and ALE images were created for each new dataset. These images were subtracted from each other and compared to the true data after 1000 permutations. A voxel-wise P value image showed the location of the true data's values on the distribution of values in that voxel. The results were thresholded with *p* = 0.01. A default cluster size > 200 mm3 was applied. Cluster analysis of contrast images uses Z score values. Surf Ice software was used to plot the resulting brain maps (https://www.nitrc.org/projects/surfice/).

## Results

### Included studies and samples characteristics

The dataset for acceptance included 8 studies with a total of 281 participants. These studies reported results for the contrast acceptance vs. no-regulation, resulting in a total of 61 foci of *increased* brain activity in acceptance. Additionally, 10 studies with a total of 364 participants reported the contrast no-regulation vs. acceptance, resulting in a total 109 foci of *decreased* brain activity in acceptance.

For reappraisal, the dataset included 24 studies with a total of 815 participants. These studies reported the contrast reappraisal vs. no-regulation, resulting in a total of 297 foci of *increased* brain activity in reappraisal. Furthermore, 8 studies with a total of 305 participants reported the contrast no-regulation vs. reappraisal, resulting in a total of 41 foci of *decreased* brain activity in reappraisal.

For completeness, the resulting ALE maps for each individual meta-analysis are presented in Supplementary Table 2 for both acceptance and reappraisal results. In the cases where uncorrected cluster-forming threshold of *p* = 0.01 did not yield significant foci (e.g., acceptance and no-regulation vs. reappraisal results), a less conservative threshold of *p* < 0.05 was used to preliminary contrast and conjunction analyses, otherwise prevented with null results on GingerAle.

### Common neural mechanisms for reappraisal and acceptance (Conjunction analysis)

**Figure 2:**
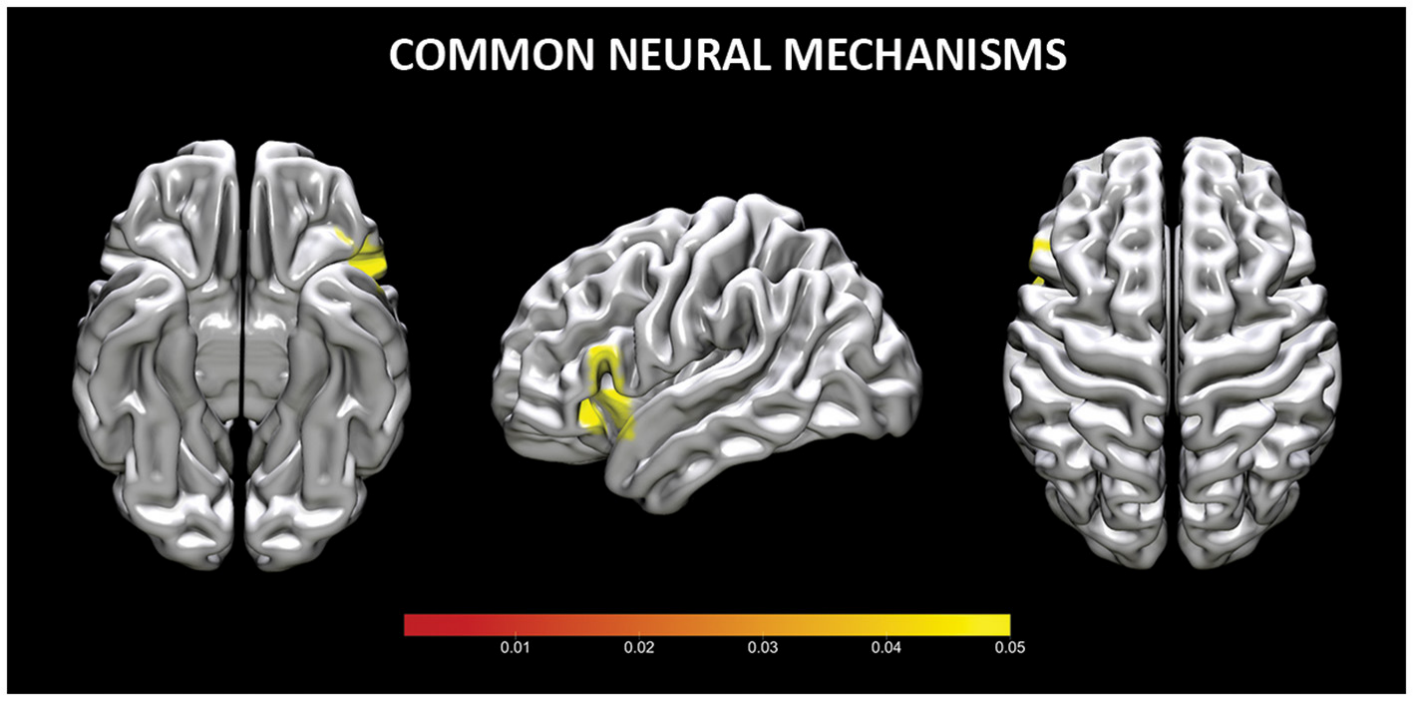
Common neural mechanisms for reappraisal and acceptance. Coordinates are reported in MNI-space.

The conjunction analysis of common increased brain activity during reappraisal and acceptance revealed three clusters of significant brain activation. Two clusters were located in the inferior frontal gyrus (or VLPFC), whereas one cluster was in VLPFC and insula (see Table 2, Figure 2). No shared clusters of decreased brain activity emerged between reappraisal and acceptance (also when results were thresholded with more lenient *p* = 0.05).

**Table 2:** Common neural mechanisms for reappraisal and acceptance. Coordinates x, y, z of local maxima refer to MNI-space.

**Cluster**	**x**	**y**	**z**	**ALE**	**Label**	**Cluster size (mm^3^)**
1	−38	24	−6	0.009	L insula	984
	−50	18	−6	0.003	L inferior frontal gyrus	
2	−50	20	12	0.002	L inferior frontal gyrus (BA 45)	24
3	−52	22	−14	0.009	L inferior frontal gyrus (BA 45)	24

BA, Brodmann Area; L, left.

### Specific neural mechanisms for reappraisal and acceptance *(Contrast analyses)*

**Figure 3:**
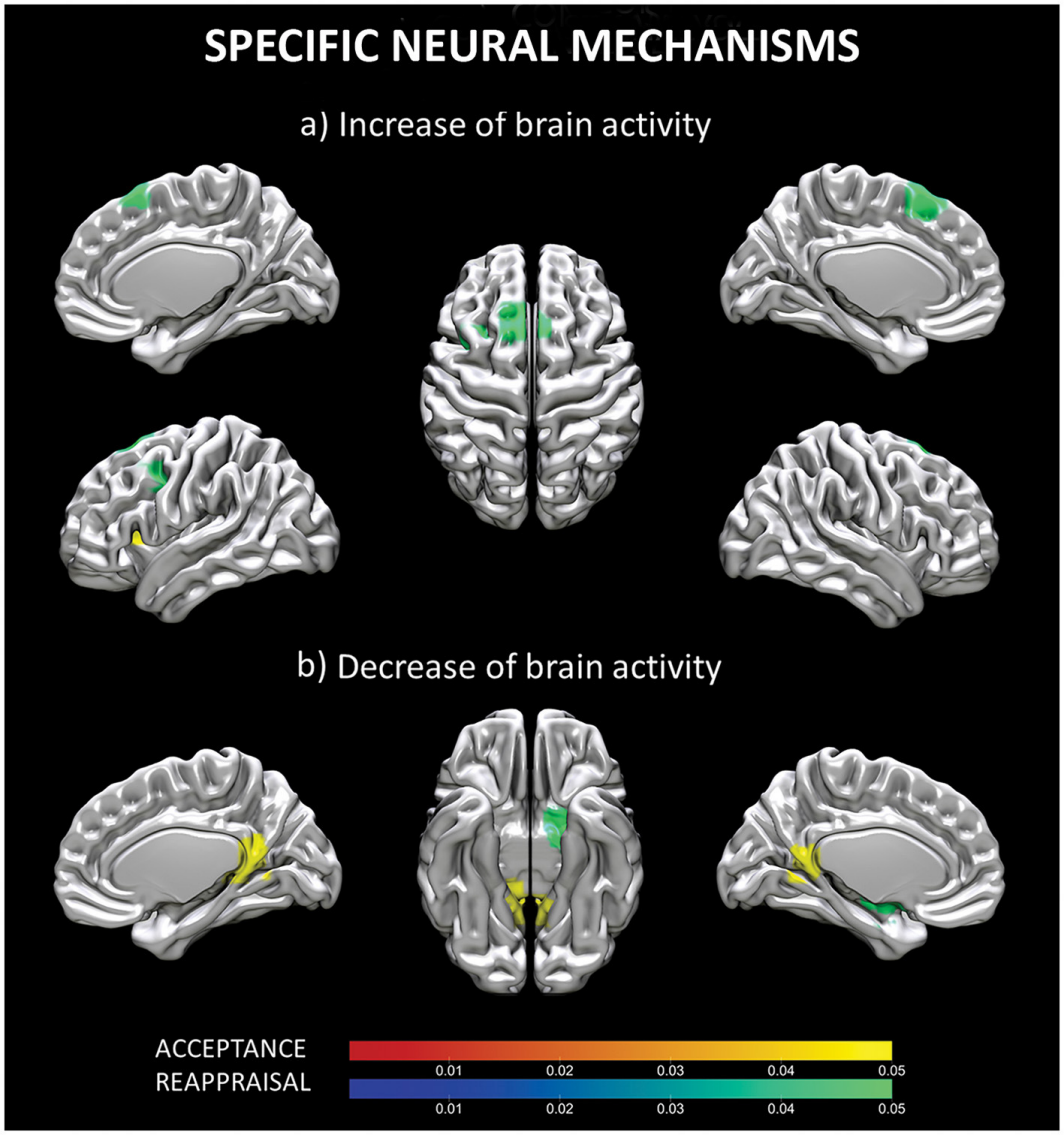
Specific neural mechanisms for reappraisal and acceptance. Increased **(a)** and decreased **(b)** brain activity for regions specifically involved in acceptance (red-yellow scale) and reappraisal (blue-green scale) strategies. Coordinates are reported in MNI-space.

When the ALE maps of reappraisal and acceptance were contrasted, two different clusters of increased and two different clusters of decreased (results thresholded with more lenient *p* = 0.05) brain activity emerged for reappraisal vs. acceptance. Increased activity was located in the superior frontal gyrus (cluster 1) and in the left middle frontal gyrus (cluster 2), whereas decrease brain activity involved left globus pallidus (cluster 1) and left putamen (cluster 2) (see Table 3, Figure 3).

**Table 3:** Specific neural mechanisms for reappraisal.

**Cluster**	**x**	**y**	**z**	***P***	**Label**	**Cluster size (mm^3^)**
**a. Increased brain activity**						
1	−14	22.3	56.3	0	L superior frontal gyrus (BA 6)	2,008
	−8.6	20.4	59.2	0.001	L superior frontal gyrus (BA 6)	
	−0.5	21	62	0.002	L superior frontal gyrus (BA 6)	
2	−34	9.5	44	0.007	L middle frontal gyrus (BA 6)	304
**b. Decrease brain activity**						
1	−16	1	−15	0.023	L globus pallidus	1,032
	−20.5	−2.7	−12.1	0.046	L globus pallidus	
2	−32	−10	−6	0.023	L putamen	240
	−27	−9.3	−9.6	0.046	L putamen	

Increased (a) and decrease (b) brain activity in the contrast analysis between reappraisal vs. acceptance. Coordinates x, y, z of local maxima refer to MNI-space. BA, Brodmann Area; L, left.

In addition, one cluster of increased and two clusters of decrease (results thresholded with more lenient *p* = 0.05) brain activity emerged as specific for acceptance vs. reappraisal. The former was located in the claustrum, whereas the latter involved bilaterally the posterior cingulate (cluster 1), the right parahippocampal gyrus, and the right thalamus (cluster 2) (see Table 4, Figure 3).

**Table 4:** Specific neural mechanisms for acceptance increased (a) and decrease (b) brain activity in the contrast analysis between acceptance vs. reappraisal.

**Cluster**	**x**	**y**	**z**	***P***	**Label**	**Cluster size (mm^3^)**
**a. Increased brain activity**						
1	−29	18	7	0.016	CLAUSTRUM	424
	−32	14	7.2	0.006	CLAUSTRUM	
**b. Decrease brain activity**						
1	11	−50	14	0.009	R posterior cingulate (BA 30)	1528
	12.9	−53	18.1	0.024	R posterior cingulate (BA 30)	
	−3.8	−52.8	9.1	0.031	L posterior cingulate (BA 30)	
2	16.4	−39.3	6.9	0.024	R parahippocampal gyrus (BA 30)	232
	18	−34	6	0.04	R pulvinar	
	11	−40	5	0.045	R parahippocampal gyrus (BA 30)	

Coordinates x. y. z of local maxima refer to MNI-space. BA, Brodmann Area; L, left; R, right.

## Discussion

Despite decades of research on emotion regulation, a comprehensive understanding of its neural basis has yet to emerged. This is partly due to the predominant focus on a subset of strategies, such as reappraisal, which has led to the misconception that a single neural substrate characterizes all emotion regulation strategies. However, in recent years, there has been a growing interest in studying a different and quite opposite set of strategies to regulate emotions, namely acceptance-based strategies (Campbell-Sills et al., 2006; Greenberg et al., 2008; Hofmann et al., 2009; Wolgast et al., 2011; Grecucci et al., 2015, 2020; Messina et al., 2021). These class of strategies have been shown to rely on different psychological compared to reappraisal-based strategies (Messina et al., 2021).

By comparing the neural bases of these two types of strategies, we can gain insights into their respective psychological mechanisms. Building on this growing body of evidence, an intriguing hypothesis is that emotion regulation processes rely on strategy-specific mechanisms that work in parallel with partially overlapping mechanisms (a core regulatory process).

To address these questions, our study conducted a meta-analysis comparing the neural underpinnings of reappraisal and acceptance strategies. The findings provide initial evidence supporting both common and distinct neural substrates for these two strategies (Grecucci et al., 2020). In the following sections, we will discuss these findings, beginning with the common core mechanism and subsequently examining the strategy-specific mechanisms. By doing so, we aim to elucidate the neural processes involved in both reappraisal and acceptance strategies.

### Common regulatory processes

The conjunction analyses in our study confirmed that both acceptance and reappraisal strategies activate common brain areas, namely the VLPFC and in the insula. The VLPFC is in various processes, including interpretating and selecting appropriate responses, inhibiting actions, and engaging in semantic and phonological processing (Morawetz et al., 2016a,b). On the other hand, the insula, plays a critical role in integrating sensory input from both the internal and external environment to shape a coherent and conscious representation of the inner emotional state (e.g., Zaki et al., 2012) and in mapping arousal associated with emotions (Grecucci et al., 2013a,b). The insula and the VLPFC have been consistently implicated in successful emotion regulation across different strategies (Morawetz et al., 2017; Li et al., 2021), including acceptance (Messina et al., 2021).

The involvement of the VLPFC aligns with recent models of emotion regulation that relativize the role of executive/controlled functions in emotion regulation and foster the importance of spontaneous, semantic, and non-effortful forms of regulation (Viviani, 2013, 2014; Messina et al., 2016). Notably, the activation of the VLPFC has been observed in other regulation processes that can be considered more implicit or non-controlled, such as emotional labeling (Tupak et al., 2014; Torre and Lieberman, 2018) and spontaneous avoidance (Viviani et al., 2010; Benelli et al., 2012). This suggests that the involvement of the VLPFC, even in the absence of core executive areas, is relevant to various forms of emotion regulation, including acceptance. These findings highlight the relevance of the VLPFC and insula in emotion regulation processes, considering their close anatomical proximity and their respective core functional roles.

In addition to the common regions activated during both reappraisal and acceptance, our preliminary data suggest that these two strategies also engage partially distinct neural regions involved in emotion regulation.

### Specific mechanisms for reappraisal

The contrast analysis confirmed that reappraisal is specifically associated with increased activity in prefrontal regions, including superior frontal gyrus or DLPFC and the middle frontal gyrus or DMPFC. This finding are consistent with previous meta-analytic studies on reappraisal (Buhle et al., 2014; Frank et al., 2014; Messina et al., 2015; Morawetz et al., 2017) that have consistently reported the involvement of these prefrontal regions. The DLPFC and DMPFC are key components of a well-established network of control-related prefrontal regions. Their engagement in reappraisal is in line with the traditional view of emotion regulation as a top-down, cognitive control process on emotions (e.g., Ochsner and Gross, 2008). In particular, the DLPFC and the DMPFC contribute to emotion regulation by facilitating response inhibition and executive control (Grecucci et al., 2013a,b; Morawetz et al., 2020). Furthermore, the recruitment of DLPFC and DMPFC is reported more prominently when reappraising highly emotional stimuli, suggesting that these regions are involved in situations that require greater cognitive resources (Morawetz et al., 2017). Not surprisingly, the same prefrontal regions underpin other top-down strategies such as distraction (Buhle et al., 2014).

Our study revealed that reappraisal is associated with decreased activity in sublobar regions, specifically the globus pallidus and putamen. These results are consistent with previous meta-analytic studies (Buhle et al., 2014; Frank et al., 2014) that also found deactivations in these regions during reappraisal, as well as increased activations during upregulation through reappraisal. The globus pallidus and putamen both belong to the basal ganglia (BG), which have traditionally been associated with motor functions. However, the basal ganglia also play a role in the Interoceptive Theory of Emotion [also known as the somatic marker hypothesis (Bechara and Damasio, 2005)]. According to this theory, emotional responses are characterized by bodily components that support the decision-making process. A recent review has emphasized the involvement of the basal ganglia in affective processing via their extensive connections with cortical and limbic regions, allowing the organism to adapt behavioral responses to emotional contexts (Pierce and Péron, 2020). The role of BG in the reinforcement learning permits the affective value (or internal state) and behavior to be shaped and applied to successive similar emotional conditions (Pierce and Péron, 2020). Therefore, decreased activity in this area can be explained as an attempt to counteract habitual emotional responses by changing the previously affective value of a given context, through reappraisal. Increased activity in the putamen has been reported in individuals with anxiety relative to healthy control (Picó-Pérez et al., 2019), suggesting its role in the network involved in cognitive action regulation (Langner et al., 2018). Connectivity analysis have also implicated the putamen and pallidum in cognitive emotion regulation (Kohn et al., 2014). These findings further support the involvement of these sublobar regions in the regulation of emotions through cognitive processes.

### Specific mechanisms for acceptance

Differently from reappraisal, our findings support the prediction that the typical network of control-related prefrontal regions is not prominently involved in the acceptance strategy. Instead, we found specific increased brain activity only in the claustrum. This area is a thin collection of neurons placed between the insular cortex and the striatum. It has been suggested to play a role in the integration of multimodal sensory input, potentially contributing to the formation of a conscious experience (Crick and Koch, 2005), thanks to the high connections with sensory modalities and cortical-subcortical neuromodulations. Alternatively, it might play a role in selective attention, especially in differentiating salient and relevant information from the irrelevant ones across different sensory modalities (Goll et al., 2015). The claustrum is thought to focus attention at a later stage of sensory processing compared to the thalamus, and, as a such, in a more selective way (Goll et al., 2015). The involvement of the claustrum in acceptance can be interpreted as an increased awareness of bodily-sensorial states (Grecucci et al., 2015; Messina et al., 2015), and as a multimodal sensory filter, allowing the excessive emotional reactivity to be minimized (Wolgast et al., 2013; Goldin et al., 2019; Dixon et al., 2020). The contribution of only this structure is consistent with previous studies that reported no detectable or reduced increased activity in prefrontal cortical areas in acceptance (Kross et al., 2009; Westbrook et al., 2013; Goldin et al., 2019; Kober et al., 2019; Dixon et al., 2020). This supports the view that acceptance is a form of regulation that does not rely on cognitive control to directly alter the emotional response (Messina et al., 2021).

The results of the acceptance-related deactivations corroborate this hypothesis. We found that acceptance is associated with a reduction in brain activity in structures of the limbic lobe, specifically the posterior cingulate cortex (PCC)/precuneus, the parahippocampal gyrus, and the thalamus (pulvinar). Interestingly, these structures are different from those specifically found for reappraisal. The PCC is a key area of the default mode network (DMN), and its deactivation may reflect the interruption of inner processes, such as rumination and mind wondering. The functional deactivation of the PCC associated with acceptance has been previously reported in another meta-analysis of acceptance studies (Messina et al., 2021). In contrast, activation of the PCC has been associated with strategies that are somewhat opposite to acceptance (based on avoidance), such as distancing (Koenigsberg et al., 2010) and distraction (Kanske et al., 2011). Notably, the PCC, and in general the DMN, are involved in semantic processing (Binder et al., 2009; Wirth et al., 2011), supporting that even in the absence of executive processes, semantic processes may serve as a form of emotion regulation.

As for PCC, the parahippocampal gyrus (PHG) has also been reported in the cluster of areas associated with acceptance (Dixon et al., 2020; Messina et al., 2021). According to some authors (Phillips et al., 2008), the PHG is part of a ventromedial neural system, as opposed to the dorsal/lateral system, and is involved in the early and automatic evaluation of the emotional meaning during emotion regulation processes. Decreased PHG connectivity has been reported during mindfulness/meditation practice (Hernández et al., 2018), while abnormal activity or connectivity in the PHG has been associated with patients with psychopathologies related to emotion dysregulation (Brown et al., 2020; Tak et al., 2021). This suggest that the reduced activity in the PHG during acceptance may reflect a reduced impact the emotional event on the individual in terms of memory association with or trace retrieval of the stimulus (Yang et al., 2017). Finally, the deactivation of the thalamus during acceptance may suggest a reduction in the filtering of sensory input, leading to increased openness and a non-judgmental attitude (Zeidan et al., 2015).

### Implications and limitations

**Figure 4:**
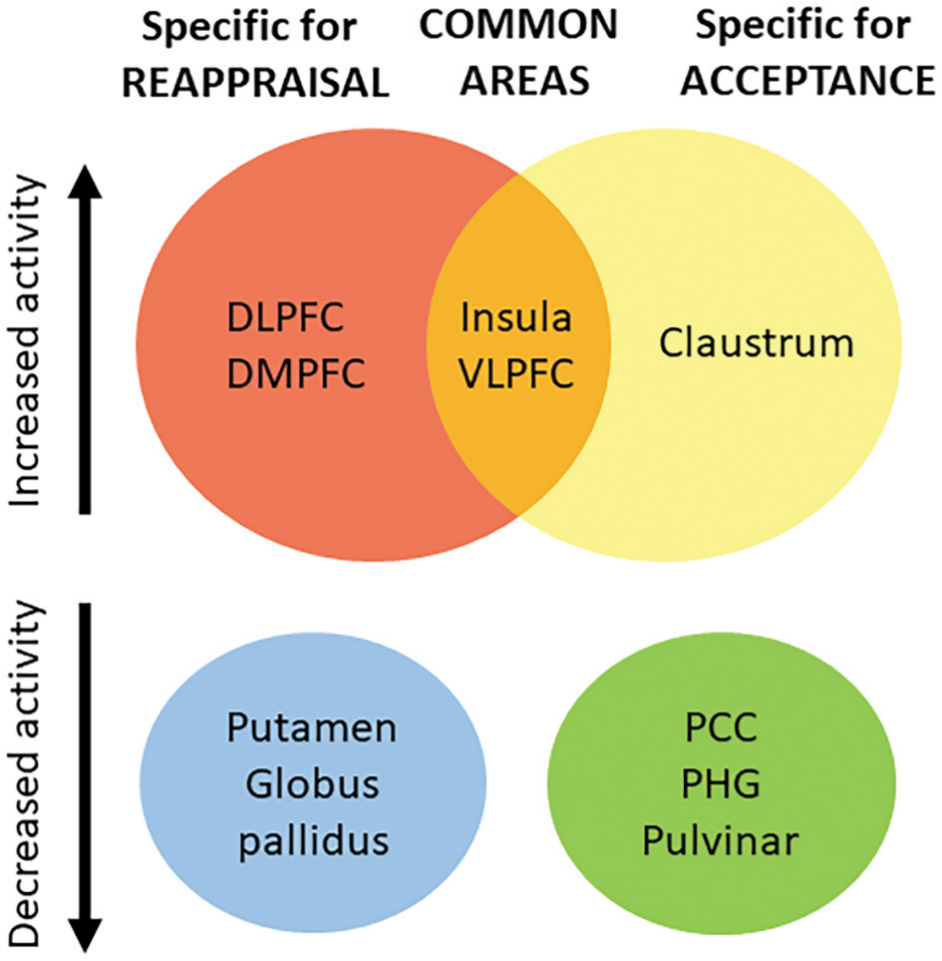
Based on our results, we suggest that emotion regulation process relies on a set of common neural areas (central part of the figure), which coexists with strategy-specific mechanisms separating reappraisal-like strategies (on the left), from acceptance-like strategies (on the right). Top of the figure: areas showing increased brain activity; Bottom of the figure: areas showing decreased brain activity. VLPFC, Ventro-lateral Prefrontal Cortex; DLPFC, Dorso-lateral Prefrontal Cortex; DMPFC, dorso-medial Prefrontal Cortex; PCC, Posterior Cingulate Cortex; PHG, Parahippocampal gyrus; IFG, Inferior Frontal Gyrus.

In this study, the results support the idea that both common and distinct mechanisms exist for reappraisal and acceptance. One implication is that previous models that consider a single cognitive model underlying all strategies (see for example, the Modal Model, Gross, 2008), or dual route models of emotion regulation (cognitive vs. experiential) (see for example, Grecucci et al., 2020), should be integrated into a more complex model. Based on our results, we suggest that emotion regulation process relies on a common neural mechanism, possibly related to a core inhibitory function (see Figure 4, central part of the figure), which coexists with strategy-specific mechanisms that separate reappraisal-like strategies (on the left), from acceptance-like strategies (on the right). Moreover, the clusters of activation and deactivation we found for the two strategies seem to be in line with a recent neural formulation that suggests the complex process of emotion regulation and emotion processing operates through the interplay of multiple large-scale neural networks, involving both cortical and subcortical regions (Morawetz et al., 2020).

Considering emotion regulation as a set of different phenomena instead of reducing it to the cognitive control of emotions has relevant clinical implications in terms of tailoring therapeutic interventions to specific clinical situations. For example, in presence of an overstated attempt to control mental content, stimulating an additional form of control using reappraisal-based therapeutic intervention may turn out to be detrimental (Purdon, 1999; Najmi and Wegner, 2008). In such cases, encouraging the adoption of a non-controlling attitude toward emotions can be more beneficial (Beevers et al., 1999; Marcks and Woods, 2005).

Another consideration is that cognitive strategies like reappraisal may not be the main choice for healthy individuals when emotion intensity is high (Sheppes et al., 2011) or when they are experiencing stress (Raio et al., 2013). Similarly, the use of reappraisal may decrease as the severity of symptom in Social Anxiety Disease increases (Goldin et al., 2009). When the deployment of cognitive resources to regulate emotion is constrained, for instance by psychopathological status, it may be a good practice using a different but still adaptive emotion regulation strategies and this should occur regardless of the limits imposed by adopting a specific approach.

Beside the merits, the finding of the present study should be considered together with the limitations, especially those concerning the samples size. Due to the novelty of the field, only an exiguous number of studies were found for acceptance. For both the strategies, in addition, the exclusive selection of the studies based on the whole-brain analyses was chosen to overcome the often pointed out limitation of inflated results due to the inclusion of ROI studies (Frank et al., 2014; see Müller et al., 2018). However, this choice had as counterpart an important reduction of the available studies (in some cases, less than the suggested 17 studies, Eickhoff et al., 2016). As the number of studies was lower than the recommended lower limits for certain contrasts, our results should be interpreted cautiously, and further studies will be crucial on this topic. Despite the small sample size, it should be noted that we decided to apply strict inclusion criteria which guarantee high homogeneity (see Müller et al., 2017 about the trade-off between power and heterogeneity). For what concerns the exclusion of ROI-based studies, this choice may have further implications for the contrast no-regulation vs. strategy. Relevant structures related to emotion processing, such as the amygdala, are typical regions of interest in task-related functional analyses. Many studies provide evidence that activity in the amygdala is dampened during emotion regulation, and such a modulation may change depending on the specific strategy adopted (e.g., Goldin et al., 2008; Ochsner et al., 2012). Unfortunately, no modulation of activity in this structure emerged in our study. This result may be explained according to the finding of a recent meta-analytic study (Gentili et al., 2019) on the neural correlates of emotional stimuli processing in phobic patients vs. healthy controls. The authors reported differences between the two groups only in the midcingulate cortex when exclusively whole-brain studies were selected. However, differences in several subcortical regions, including the amygdala, emerged when ROI-based studies were also included. Finally, although we acknowledge that a more stringent uncorrected cluster-forming threshold is commonly used in meta-analytic studies (Müller et al., 2018), we also agree that this threshold is conventionally chosen and “any other uncorrected voxel-wise thresholds would also be perfectly valid” (Eickhoff et al., 2012, pg. 2353-2354). We hope that our preliminary, yet promising finding will stimulate further neuroscientific investigations on emotion-focused strategies, leading to a larger sample size, and allowing future metanalytic comparisons to apply more stringent parameters.

## Conclusions

Reappraisal and acceptance are different effective processes for regulating emotions in response to distressing events (Aldao et al., 2010; Kohl et al., 2012; McRae, 2016; Grecucci et al., 2020). In clinical psychology, the usefulness of such strategies is debated and different views concerning the usefulness of reappraisal to control emotion exist along with the adoption of acceptance/non-controlling attitude toward emotions (Hofmann et al., 2009; Wolgast et al., 2011; Diedrich et al., 2016; Frederickson et al., 2018). With the present meta-analytic study, our aim was to contribute to this debate by shedding new light on the nature of common and specific patterns of brain activity associated with these processes. We believe that by comparing these opposite strategies, the neural architecture of emotion regulation processes can be better outlined, by an exhaustive description of its various facet.

## Data availability statement

The original contributions presented in the study are included in the article/Supplementary material, further inquiries can be directed to the corresponding author.

## Author contributions

BM: formal analysis and writing—original draft. AG and IM: conceptualization, supervision, and writing—review and editing. PA: writing—review and editing. All authors contributed to the article and approved the submitted version.
