# Supplementary material for: Comparing reappraisal and acceptance strategies to understand the neural architecture of emotion regulation: a meta-analytic approach
Source: Front Psychol. 2023 Jul 21;14:1187092. doi: 10.3389/fpsyg.2023.1187092 (PMC10403290; doi:10.3389/fpsyg.2023.1187092)
Supplement: Supplementary file 2 [file Table_2.DOCX]

**Supplementary material**

**Table S2. Increased (a) and decreased (b) brain activity in the contrast analysis for acceptance (restricted analysis). Coordinates x, y, z of local maxima refer to MNI-space. BA = Brodmann Area; L= left; R = Right.**

| **Cluster** | **x** | **y** | **z** | **ALE** | **P** | **Z** | **Label** |
| --- | --- | --- | --- | --- | --- | --- | --- |
| **a. Increased brain activity (restricted analysis)** | | | | | | | |
| 1 | -36 | 26 | -4 | 0.012 | <0.001 | 4.055 | L insula |
|  | -44 | 8 | 6 | 0.009 | <0.001 | 3.523 | L precentral gyrus (BA 44) |
|  | -32 | 16 | 12 | 0.009 | <0.001 | 3.287 | L insula (BA 13) |
|  | -52 | 16 | 16 | 0.009 | <0.001 | 3.287 | L inferior frontal gyrus (BA 44) |
|  | -52 | 16 | -6 | 0.009 | <0.001 | 3.287 | L inferior frontal gyrus (BA 47) |
| **b. Decreased brain activity (restricted analysis)** | | | | | | | |
| 1 | -4 | -54 | 6 | 0.013 | <0.001 | 3.986 | L culmen |
|  | -6 | -56 | 26 | 0.012 | <0.001 | 3.830 | L posterior cingulate (BA 31) |
|  | -10 | -32 | 6 | 0.010 | <0.001 | 3.465 | L thalamus (Pulvinar) |
|  | -18 | -34 | 14 | 0.010 | <0.001 | 3.378 | L thalamus (Pulvinar) |
|  | 0 | -34 | 2 | 0.010 | <0.001 | 3.358 | lingual gyrus (BA 27) |
|  | 6 | -52 | 12 | 0.009 | <0.001 | 3.146 | R posterior cingulate (BA 29) |
|  | -12 | -44 | -2 | 0.009 | <0.001 | 3.141 | L parahippocampal gyrus (BA 30) |
|  | 14 | -38 | 4 | 0.009 | <0.001 | 3.111 | R parahippocampal gyrus (BA 30) |
|  | 24 | -34 | 2 | 0.009 | <0.001 | 3.094 | R thalamus (Pulvinar) |
|  | -12 | -72 | 18 | 0.009 | 0.001 | 3.081 | L cuneus (BA 18) |
|  | 10 | -54 | 18 | 0.009 | 0.001 | 3.070 | R posterior cingulate (BA 30) |
|  | -38 | -14 | 12 | 0.008 | 0.001 | 3.061 | L insula (BA 13) |
|  | -8 | -70 | 6 | 0.008 | 0.001 | 3.031 | L lingual gyrus |
|  | -24 | -22 | 10 | 0.008 | 0.002 | 2.965 | L thalamus |
|  | 2 | -46 | 0 | 0.008 | 0.002 | 2.880 | L culmen |

**Table S3. Increased (a) and decreased (b) brain activity in the contrast analysis for reappraisal. Coordinates x, y, z of local maxima refer to MNI-space. BA = Brodmann Area; L= left; R = Right.**

| **Cluster** | **x** | **y** | **z** | **ALE** | **P** | **Z** | **Label** |
| --- | --- | --- | --- | --- | --- | --- | --- |
| **a. Increased brain activity** | | | | | | | |
| 1 | -6 | 10 | 60 | 0.030 | <0.001 | 5.67 | L Medial Frontal Gyrus (BA 6) |
| 1 | 16 | 20 | 60 | 0.020 | <0.001 | 4.30 | R Superior Frontal Gyrus (BA 6) |
| 1 | 10 | 28 | 44 | 0.015 | <0.001 | 3.50 | R Medial Frontal Gyrus (BA 8) |
| 1 | 12 | 22 | 36 | 0.010 | 0.004 | 2.66 | R Cingulate Gyrus (BA 32) |
| 2 | -48 | 32 | -8 | 0.033 | <0.001 | 6.03 | L Inferior Frontal Gyrus (BA 45) |
| 2 | -56 | 28 | 8 | 0.016 | <0.001 | 3.70 | L Inferior Frontal Gyrus (BA 45) |
| 2 | -50 | 26 | 2 | 0.014 | <0.001 | 3.34 | L Inferior Frontal Gyrus (BA 45) |
| 2 | -50 | 24 | 12 | 0.013 | 0.001 | 3.19 | L Inferior Frontal Gyrus (BA 45) |
| 3 | -40 | 12 | 56 | 0.020 | <0.001 | 4.23 | L Middle Frontal Gyrus (BA 6) |
| 3 | -36 | 14 | 40 | 0.017 | <0.001 | 3.88 | L Middle Frontal Gyrus (BA 6) |
| 3 | -34 | 20 | 46 | 0.014 | <0.001 | 3.40 | L Middle Frontal Gyrus (BA 6) |
| 3 | -36 | 8 | 42 | 0.014 | <0.001 | 3.37 | L Middle Frontal Gyrus (BA 6) |
| 3 | -48 | 16 | 42 | 0.010 | 0.004 | 2.62 | L Middle Frontal Gyrus (BA 8) |
| 4 | -46 | -64 | 30 | 0.019 | <0.001 | 4.14 | L Middle Temporal Gyrus (BA 39) |
| 4 | -40 | -56 | 22 | 0.013 | 0.001 | 3.19 | L Superior Temporal Gyrus (BA 22) |
| 4 | -52 | -62 | 42 | 0.013 | 0.001 | 3.10 | L Inferior Parietal Lobule (BA 39) |
| 5 | -54 | -38 | 0 | 0.028 | <0.001 | 5.42 | L Middle Temporal Gyrus (BA 22) |
| **b. Decreased brain activity** | | | | | | | |
| 1 | 2 | 14 | -6 | 0.012 | <0.001 | 4.150 | R Anterior Cingulate (BA 25) |
| 1 | -28 | -4 | -8 | 0.011 | <0.001 | 4.040 | L Putamen |
| 1 | 2 | 24 | -20 | 0.011 | <0.001 | 3.736 | R Medial Frontal Gyrus (BA 25) |
| 1 | -22 | -2 | -16 | 0.009 | 0.001 | 3.260 | L Parahippocampal Gyrus (BA 34) |
| 1 | -16 | -6 | -8 | 0.008 | 0.001 | 3.217 | L Medial Globus Pallidus |
| 2 | 6 | 72 | 2 | 0.011 | <0.001 | 3.736 | R Medial Frontal Gyrus (BA 10) |
| 2 | 2 | 64 | -8 | 0.011 | <0.001 | 3.736 | R Frontal Lobe |
| 2 | 2 | 62 | -20 | 0.011 | <0.001 | 3.736 | R Medial Frontal Gyrus (BA 10) |
